# OECI-EACR precision medicine for cancer: Conference report 1–4 March 2015, Luxembourg

**DOI:** 10.3332/ecancer.2015.519

**Published:** 2015-04-03

**Authors:** Anna Golebiewska, Sabrina Fritah, Maria Romina Girotti

**Affiliations:** 1 NORLUX Neuro-Oncology Laboratory, Department of Oncology, Luxembourg Institute of Health (L.I.H), Luxembourg, L-1526 Luxembourg; 2 Molecular Oncology Group, Cancer Research UK Manchester Institute, Manchester, M20 4BX, UK

**Keywords:** OECI-EACR 2015, conference report, precision medicine, cancer, personalised medicine

## Abstract

The ‘Precision Medicine for Cancer’ was the first meeting of a new series of conferences organised biannually by the European Association for Cancer Research (EACR) and the Organisation for European Cancer Institutes (OECI). The main objective of the meeting was to focus on novel topics in precision medicine by allowing strong interactions between participants and to access the speakers easily. As the first implementations of personalised medicine are appreciated in the clinic, the aim of the meeting was to further educate both researchers and clinicians and learn more from the novel approaches in the field. Similarly, the interaction between two organisations—the research-oriented EACR and the clinic-oriented OECI—was of a great value for the meeting. This OECI-EACR 2015 report will highlight the major findings of this outstanding meeting.

The conference took place in Luxembourg, a place known more as a financial center rather than a research site. The conference started with the welcoming words from the organisers: **Professor Simone Niclou**, the local organizer from the Luxembourg Institute of Health, the head of the NorLux Neuro Oncology laboratory; **Professor Richard Marais**, the head of the Molecular Oncology Lab, the Director of the CRUK Manchester Institute and the EACR President; and **Professor Giorgio Stanta** from the University of Trieste (Italy), representing the OECI. They all stated the aims of the conference: educating people and scientists in the new era of personalized medicine.

During recent years, as part of socio-economic changes in the country the Luxembourgish government established a strategic plan to invest in the fundamental and applied research and to put Luxembourg on the map of European research centers. The National Research Fund (Fonds National de la Recherche, FNR) is the key player in establishing a high-quality research environment in Luxembourg. The strategic objectives of the organisation were presented during the conference by **Dr Frank Glod**, the head of the FNR’s ‘Strategic Research Programmes’ unit, which include funding national and international research projects, promoting scientific culture and interest in research in the society.

The conference was followed by scientific talks from lead experts in the field of personalised medicine.

## Session 1: Personalising precision medicine

The opening session of the conference focused on novel approaches and main challenges in translating precision medicine protocols in the clinic.

A gold standard for testing novel therapeutical protocols involves randomised controlled clinical trials, however the precision medicine should also make a use of information from existing treatments and epidemiology. **Professor Thomas A. Sellers,** the director of the Moffitt Cancer Centre and Research Institute in Tampa, Florida (USA), featured the opening keynote lecture. Professor Seller is a worldwide expert in molecular and genetic epidemiology and he aims to use the existing knowledge to predict risk and response to current therapies. In his opinion much can be learned outside of the clinical trials form the existing information, including somatic and germline variants, pathological and surgical parameters and lifestyle of the patients. The precision medicine protocols are designed not only to increase therapeutic outcome but also to decrease cost on health care. During the lecture we have learned about the remarkable effort made by the Moffitt Cancer Center in establishing the outstanding infrastructure for the research protocols. Importantly, as highlighted by the speaker, the platform was created not only for the clinicians and researchers but also is of a great use for the hospital administration and patients themselves. The data includes patient information and consent, health records, tissue collection, and data maintenance. Even more impressive, the Moffitt Cancer Center further expands the collection of patient information with the other cancer centres in USA within the Oncology Research Information Exchange Network (ORIEN) in hope to accelerate the development of targeted therapies and match patients more appropriately to the ongoing clinical trials. The two following talks of the session focused on the importance of the tumour heterogeneity in designing novel therapies and understanding drug resistance mechanisms. **Professor Simone Niclou,** the head of Norlux Neuro-Oncology laboratory at the Luxembourg Institute of Health (L.I.H), summarized our current understanding on very strong inter- and intra-tumoural heterogeneity in glioblastoma, the most aggressive and incurable type of brain tumours. She presented current attempts to stratify the glioblastoma patients based on genetic profile, transcriptome, epigenome and altered molecular pathways. Within the adult glioblastomas the integrative analysis at different levels revealed so far only one clear subgroup of patients with IDH1 mutation associated with hypermethylated phenotype and better survival prognosis. Unfortunately all recent clinical trials failed and more effort is needed to better understand this very heterogeneous and rare disease. The additional challenge in designing novel treatments lies in the strong intertumoural heterogeneity. Evolution of tumours in time together with regional heterogeneity needs to be evaluated in more detail in the future. Professor Niclou highlighted the novel data from her group showing very strong inter- and intratumoural heterogeneity at the ploidy level [37], where although aneuploidization appears as late event in tumour development it gives rise to more aggressive clones. Moreover, she suggested that cancer stem cells might not be an appropriate target for therapies as current cancer stem cell markers not only are not specific for tumour cells [16] but also display very heterogeneous profile in different patients and do not define a uniform genetic clone [37]. A strong tumour heterogeneity is also a main burden responsible for failure of current therapies in colorectal cancer. **Professor Alberto Bardelli** from the Candiolo Cancer Institute at the University of Torino (Italy) presented recent data on how his group endeavours to define relationship between genotype and response to drug treatment in colorectal cancer. Importantly colorectal cancers represent molecularly distinct diseases, therefore personalised protocols are needed to improve therapeutic outcomes. The strong genetic heterogeneity is present not only between patients but also between multiple metastatic sites of the same patient and even within the same lesion. Professor Bardelli focused particularly on the primary and secondary resistance to EGFR-targeted therapies that remarkably are driven by the mutations in the same genes and lead to activation of the same key signalling pathways (MEK/ ERK activation) [25, 26]. He highlighted the potential of monitoring the occurrence of novel mutations in plasma samples from patients who developed resistance to anti-EGFR antibodies and recapitulation of treatment regime in patient-derived xenografts models [24]. He introduced the concept of liquid biopsies as the use of blood to understand the complexity of the disease because interrogating one single lesion is not sufficient. In other words, the use of blood to study the genetics of cancer.

## Session 2: Tumour heterogeneity

The second session of the conference focused mostly on the current challenges and heterogeneity in breast cancer. **Professor Anne-Lise Børresen-Dale** from the Institute for Cancer Research at the Oslo University Hospital (Norway) presented a remarkable effort of her group in assessing tumour heterogeneity with use of numerous high-throughput techniques [22]. The combined analysis of DNA copy-number alterations, DNA methylation, mRNA and miRNA profiling, proteomic expression, and metabolomics analysis can support superior subclassification of breast cancers for personalised therapies and find novel prognostic factors. However bringing all the data together is a challenge and requires strong interdisciplinary effort [32]. We have also learned about the power of quantitative ‘double immunoFISH’ technique (immunostaining combined with FISH) for detailed analysis of intratumoural heterogeneity with patient specific markers. The strong genomic heterogeneity was shown to be responsible for lower response to therapies and stickily tumours after treatment appeared even more heterogeneous. Single cell analysis revealed further than breast tumours undergo constant clonal evolution and numerous metastatic sites appear to derive from the lymph node metastasis rather than initial tumour itself [27]. **Professor Carlos Caldas** from the Cancer Research UK Cambridge Institute (UK) presented novel classification of breast cancers into 10 genomic-driven based subtypes. Breast cancer is mainly driven by the somatic copy number alterations which strongly correlate to gene expression [6]. IntClust classification is defined by distinct genomic and transcriptomic landscapes and appears as a very robust and reproducible method [2]. IntClusts are associated with distinct clinical outcomes, including relapse-free survival and chemosensitivity [8]. Hopefully the subclassification of patients will better define the driver mutations and allocate the best personalised treatment for each patient. Professor Caldas group is currently developing patient-derived xenografts that would represent the 10 patient subtypes, which can be used for extensive drug screens where drug responses can be directly correlated to molecular profiles. Interestingly, although patient-derived xenografts show similar patterns to patients, tumours can undergo reproducible clonal selection and evolution in animals [9]. This will be of a great value for future studies predicting patient response with use of matched xenografts models. The first proffered paper was presented by **Dr Jerome Paggetti** from the Laboratory of Experimental Hemato-Oncology at the Luxembourg Institute of Health (L.I.H.) focused on the role of exosomes in communication between tumour and stromal cells. Exosomes are small extracellular vesicles originated from tumour endosomes that carry small RNAs (miRNA, miscRNAs, oncomiRs), mRNA, DNA and proteins. Circulating miRNAs are sensitive prognostic markers for chronic lymphocytic leukemia (CLL) [30, 28] and exosomes derived from CLL tumour cells can actively enter stromal endothelial and mesenchymal stem cells and activate key signalling pathways and secretion of inflammatory cytokines and angiogenesis. As a feedback mechanism, exosome-modified stromal cells stimulate CLL tumour cells to promote leukemogenesis. This highlights importance of stromal compartment is shaping the disease progression and exosomes should be considered not only as a biomarker but also as a potential therapeutic target in CLL.

## Session 3: Responses to precision drugs

**Professor Manel Esteller** from the The Bellvitge Institute for Biomedical Research (IDIBELL) in Barcelona (Spain) showed us that both in health and disease, DNA methylation is a key determinant of the epigenome. Professor Manel Esteller recapitulated the essential contribution of DNA methylation in the evolution of pharmacoepigenetics accross different cancer types. In glioma the promoter methylation of the DNA repair enzyme (O(6)-Methylguanine-DNA methyltransferase (MGMT) is a prognostic marker of patient response to Temozolomide currently used in the clinics. This marker also predicts the clinical response to dacarbazine in metastatic colorectal cancer [3]. In ovarian and breast cancer, BRCA1 promoter methylation reflects the sensitivity to cisplatin [36]. Outside promoter methylation, the genetic defects in epigenetic genes confer resistance to epigenetic and classical drugs. Finally, using advanced technology of whole genome bisulfite sequencing, a dynamic DNA methylation of promoter and genes bodies is associated to the epithelial to mesenchymal transition in cancer [4]. Of interest, DNA methlylation fingerprints are developped to identify the type origin of cancer of unknown primary origin. Later on, **Professor Jukka Westermarck** from the Institute of Molecular Medicine at University of Helsinki (Finland) presented the second proffered paper. His results showed that PP2A is a major determinant of therapeutic response in cancer cells and its inactivation is linked to a poor prognosis in about 12 cancer types. PP2A is inhibited by the oncoprotein CIP2A, Cancerous Inhibitor of Protein phosphatase 2A [21]. In head and neck squamous cell cancer, CIP2A regulates oncogenic and radioresistance properties and therefore represents a valuable therapeutic target [39]. **Professor Ultan McDermott** from the Sanger Institute in Cambirdge (UK) presented how use of large scale sequencing generated through the International Cancer Genome Consortium and The Cancer Genome Atlas allows the current definition of cancer genome patterns. These impressive resources provide a catalogue of mutational processes and genomic alterations for pancancer and cancer specific analyses [1]. A second powerful experimental design is the ongoing construction of living organoid biobank of cancers and the development of genetic screens (CRISPR) for essential gene identification [11]. Synthetic lethality screens will help our understanding on the impact of tumour genomes in drug responses. Exploring cancer biology using big data and relevant models will hopefully accelerate the identification of biomarkers and actionable genes for drug targets. This session was closed by **Professor Stefan Pfister** from the German Cancer Research Centre (DFKZ) in Heidelberg (Germany). Unlike many others cancers, clinical decisions for patients with brain tumours rely mainly on histopathology which should, in the future be complemented with molecular informations for accurate diagostics [20]. We have learned from Professor Pfister how addtional information from DNA methylation revolutionizes tumour subgrouping in pediatric neurooncology, particularly in medulloblastoma and ependymoma [10]. The impressive implemenation of (epi)genomics data in clinical decisions is ongoing through the individualized therapy for relapsed malignancies in the childhood (INFORM) consortium.

## Session 4: Precision Medicine in Melanoma

This session featured leaders in the field of melanoma presenting their advances in precision medicine for this disease. The session started with **Professor Richard Marais,** the director of the CRUK Manchester Institute (UK) and the EACR President. He showed us how the history of melanoma treatment has changed since the discovery of BRAF as an oncogene in cancer [7]. New targeted therapies developed to target BRAFv600 achieve impressive clinical results in melanoma patients but the development of resistance seems inevitable in most cases [14, 15]. Moreover, BRAF inhibitors drive RAS-dependent BRAF binding to CRAF, CRAF activation, and MEK-ERK signaling in oncogenic RAS [18] revealing a paradigm of BRAF-mediated signaling that promotes tumour progression with clinical implications [38] and highlighting the importance of understanding pathway signaling in clinical practice and of genotyping tumours prior to administering BRAF-selective drugs, to identify patients who are likely to respond and also to identify patients who may experience adverse effects such as increased invasion [33]. Importantly, there is hope for patients with resistant melanoma. Two novel panRAF inhibitors with anti SRC activity were developed as part of a drug discovery program and are scheduled to enter a clinical trial this year [13]. Then **Dr Maria Romina Girotti** from the Molecular Oncology Lab at the CRUK Manchester Institute (UK) presented the third proferred paper where she descirbed a new platfrom for personalised medicine in melanoma patients. Dr Girotti showed that BRAF inhibitors such as vemurafenib or dabrafenib, or MEK inhibitors, trametinib and cobimetinib, block the RAS-RAF-MEK-ERK pathway, inhibiting cell growth and increasing progression-free and overall survival in patients whose tumours carry BRAF mutations. However, responses are generally limited and after a relatively short period (6-8 months) of disease control, most patients develop resistance. Selecting second line therapies is challenging and current advice includes an option to continue targeted treatment beyond progression. To develop approaches improving patient outcomes with targeted therapies she described the importance of integrating several methodologies, including whole exome sequencing (WES), patient derived xenografts (PDX), longitudinal analysis of circulating tumour DNA (ctDNA) as a liquid biopsy from melanoma patients on standard chemotherapy and targeted therapies. The session was followed by **Professor Daniel Peeper** from the Netherlands Cancer Institute (NKI) in Amsterdam. Professor Pepper presented importance of parallel in vivo and in vitro negative-selection short hairpin RNA (shRNA) screens for genes that preferentially contribute to tumour cell proliferation and survival in vivo in melanoma. He found that melanoma cells harboring shRNAs targeting several DNA damage response (DDR) kinases had a greater selective disadvantage in vivo than in vitro, indicating an essential contribution of these factors during tumour expansion. DDR kinases became activated following hypoxia and either the depletion or pharmacologic inhibition of DDR kinases were toxic to melanoma cells. This included resistance to the BRAF inhibitor, and this could be enhanced by angiogenesis blockade. These results reveal that hypoxia sensitizes melanomas to targeted inhibition of the DDR showing the utility of in vivo shRNA dropout screens for the identification of pharmacologically tractable targets [31]. **Professor Jeffrey Sosman** from the Vanderbilt University Medical Center in Nashville (USA), focused on BRAF wild type melanomas. His approach has centered on finding ways to better target NRAS mutant melanoma and better define subsets in the NRAS, cohort that may be responsive to specific therapies. Preclinical experiments have shown a greatly enhanced anti-tumour response when CDK4/6 inhibitors were added to MEK inhibitors [23]. This finding along with TCGA data that demonstrated a high frequency of alterations in the genes regulating cell cycle led to two phase I/II clinical trials combining MEK inhibitor (binimetinib or trametinib) with CDK4/6 inhibitors (LEE001 or palbociclib). The early results in the trial combining binimetinib and LEE001 demonstrate clinically significant objective responses in 7/21 (33%) patients with most of the remaining patients demonstrating tumour reduction. Professor Sosman also commented on the existence of atypical BRAF mutations (non-V600) and BRAF fusions activating the MAP kinase pathway in about 10% of the BRAF negative and NRAS negative population. In vivo studies demonstrate the sensitivity of these genetic alterations to MEK inhibitors and this is currently explored clinically.

## Session 5: Mining the metabolome for precision drug targets

**Professor Susan Critchlow** from AstraZeneca in the UK presented metabolic reprogramming in cancer and novel drugs targeting cancer metabolism. The metabolic phenotype of many tumours switches from oxidative phosphorylation to aerobic (Warburg Effect) or anaerobic glycolysis increasing the rates of glucose consumption and lactate production enabling tumours to meet their energy and biosynthetic demands even under conditions of low nutrients and O2. The final product of glycolysis, lactate, is a metabolic ‘dead-end’, which if allowed to accumulate in the tumour cell can cause feedback inhibition of glycolysis, intra-cellular acidification and inhibition of cell growth. Therefore pharmacological inhibition of lactate transport represents a promising therapeutic strategy to target a range of human cancers. Monocarboxylate transporters (MCT1-4) catalyse proton-linked transport of monocarboxylates across the plasma membrane with MCT1 and MCT4 being the key tumour-associated lactate transporters. AZ has developed a new drug AZD3965 which targets MCT1, representing the first class small molecule inhibitor targeting glycolytic phenotype of tumours with great antitumour effects in renal cell carcinoma. **Professor Eyal Gottlieb** from the CRUK Beatson Institute in Glasgow (UK), focused on metabolic adaptation of cancer cells to changing environments and stress. We have learned that fatty acid synthase (FASH) is over-expressed in numerous cancers, presumably because there is a limited source of fatty acids for growth. Cancer cells are sensitive to FASH inhibitors upon stress conditions (low serum). Compared to normoxia, upon hypoxia fatty acids present reduction in polysaturated lipids indicating limited de novo fatty acid synthesis. Interestingly, although 13C carbon incorporation from glucose is reduced in hypoxia the total amount of lipids is higher, suggesting use of another carbon source by hypoxia-stressed cells. Acetyl-coA synthetase 2 (ACSS2) is focally amplified in breast cancer and correlates with poor prognosis. ACSS2, which uses acetate to produce Acetyl-CoA-a key metabolic hub- was found to be essential for survival upon metabolic stress and in vivo. Characterization of acetate metabolism confirmed it was consumed by cancer cells under hypoxia in an ACSS2-dependent manner and was used to synthesize lipids and TCA-cycle intermediates [34]. Interestingly, the results revealed a role for acetate as a nutritional source for the growth and survival of cancer cells under metabolically stressful conditions. The fourth proffered paper presented by **Dr Bassam Janji** from the Laboratory of Experimental Hemato-Oncology at the Luxembourg Institute of Health (L.I.H.) focused on the role of autophagy in regulating anti-tumour immune response in hypoxia. Hypoxia, a feature of most solid tumours, is known to influence different aspects of tumour biology including metabolism and contribute strongly to chemo- and radioresistance. Hypoxia is also involved in immunosuppression, an important drawback for targeted immunotherapies. Dr Janji stressed that even under active immune response, tumour cells escape immunosurveillance under hypoxic stress. Hypoxic tumour cells can escape both the cytotoxic T lymphocyte (CTL)-mediated adaptive response [29] and the NK cell-mediate innate immune response [40] by activating autophagy. Therefore inhibition of autophagy might be crucial to improve tumour cell killing mediated by immune system in highly hypoxic tumours.

## Session 6: New Strategies

This session started with the fifth proferred paper presented by **Dr Zhi Xiong Chen** from the National University of Singapore. Number of neuroblastoma (20–30%) carry a deletion of 1p36 on which is located KIF1Bb, a possible tumour suppressor gene. The work of Dr Chen provides some molecular basis of this mechanism. KIF1Bb interacts with DHX9 and is required for KIF1Bb-mediated apoptosis. This complex regulates the level of the apoptotic gene XAF1 [5]. As a result, XAF1 expression impact on neuroblastoma tumour growth, could therefore represent a rationale therapeutic target. Patient-derived xenograft models are superior compared to classical long-term in vitro cultures as they recapitulate closely the genetic and phenotypic features of patient tumors. **Dr Anna Golebiewska** from the Norlux Neuro-Oncology laboratory at the Luxembourg Institute of Health (L.I.H) presented the sixth proffered paper. Her talk touched on the glioblastoma xenograft model based on organotypic spheroids. The xenografts derived in eGFP expressing NOD/Scid mice allow appropriate discrimination between tumour and stromal cells and in detail characterisation of the two compartments without manipulation of the patient-derived material [16, 17]. Importantly, the glioblastoma xenografts recapitulate histopathological heterogeneity and tumours can be stratified into invasive, intermediate and angiogenic phenotypes, based on the presence and intensity of the classical histopathological features and the molecular profile of tumour cells. Interestingly tumour cells of diverse phenotypes establish different stromal compartments and differentially activate stromal endothelial cells. This shows importance of stromal compartment not only for intra- but also inter-tumoural heterogeneity. The session was closed by **Professor Sergio Quezada** from the UCL cancer Institute at the University College London (UK). The intra-tumour balance of immune cells is dynamic upon tumour growth and evolves towards a low ratio between effective and regulatory T cells. The outcome of this homeostasis is of utmost importance for survival and often results in the immune control escape of tumour cells. Immunomodulatory strategies using antibodies against CTLA-4 modify this balance and increase the proportion of effective T lymphocytes. The work presented by Professor Quezada investigated this therapeutic strategy in a murine model of melanoma. His results showed that the expression of FcgRIV on myeloid cells expressing CD11b is required for intratumoural T reg depletion [35]. Specific features of the microenvironment is decisive for the outcome of immuno modulatory strategies using antibodies.

## Session 7: Resistance to precision medicines

**Professor Passi Janne,** director of the Lowe Center for Thoracic Oncology and Scientific Director of the Belfer Institute for Applied Cancer Science in Boston (USA) communicated on EGFR and resistance in SCLC. The most common mechanism of resistance involves mutation of the target. This results in pathway reactivation, so new drugs or new drug combinations are needed. Importantly, the therapy should be both effective and tolerable. The most common mutation in lung cancer is T790M in EGFR. Alternative/covalent EGFR inhibitor should overcome increased affinity to ATP in the EGFR T790M. Unfortunately afatinib, a covalent EGFR inhibitor, failed in clinic due to very high effective concentrations which led to too strong toxicity. By using a cell line model with mutation and a screen for novel class of inhibitors Professor Janne found agents that were ~100X more potent. Not only were they more active, they were also mutation selective. There are currently many next generation drugs in the pipeline for single and combinational studies; two have received advanced drug status: AZD9291 and WZ4002. AZD9291 trial resulted in 50% objective responses (tumour responses) and more importantly due to acceptable IC50 for both EGFR wt and T790M it can be used further for combinational studies. WZ4002, another covalent EGFR T790M inhibitor, while combined with trametinib, a MEK inhibitor, prevents emergence of resistance from the onset of the treatment. **Professor Caroline Dive** from the CRUK Manchester Institute (UK), presented exciting results about the role of Circulating Tumour Cells (CTCs) in small-cell lung cancer (SCLC). This is an aggressive tumour with early dissemination and bad prognosis, and biopsies to investigate SCLC biology are difficult to obtain. Professor Dive focused on the function and potential biomarker role of CTCs, which are prevalent in SCLC, and present another ‘liquid biopsy’ option. CTCs in SCLC can be used not only as a prognostic marker but also as a potential predictive biomarker. Importantly, CTCs from patients with either chemosensitive or chemorefractory SCLC are tumourigenic in immune-compromised mice, and the resultant CTC-derived xenografts (CDXs) mirror the patient’s response to chemotherapy. The genetic characterization of CTCs and CDX showed considerable similarity [19]. She proposed that serial blood sampling could facilitate delivery of personalized medicine for SCLC. Nevertheless, analysis of CTCs in blood is still very challenging and more precise novel technologies are needed for the clinic. **Dr Safia Thaminy** presented the seventh proffered paper from the ETH in Zurich (Switzerland) and the Institute for Systems Biology, Seattle (USA). She studied the role of hypoxia in breast cancer and used breast cancer cell lines with distinct aggressiveness properties to develope an innovative strategy based on a system-wide quantitative proteomics in combination with a high-throughput migration screen and protein network analysis. Dr Thaminy found that the less aggressive cell line temporally regulated cell migration under hypoxia and the underlying mechanism involved the PTPRG and TACSTD2 driver genes. In a large-scale migration screen, stathmin (STMN1) remarkably discriminated the poorly invasive from the highly invasive cell line and STMN1 knockdown dramatically enhanced cell migration of poorly invasive cells only under hypoxia. With these results she proposed a model of cancer progression based on the hypoxia-dependent trafficking of vesicles and their release in the extracellular space. Finally **Professor Anton Berns** from the Netherlands Cancer Institute (NKI) in Amsterdam presented the closing keynote lecture. Professor Berns is a world leader in creating genetically engineered mouse models for analysis of tumour initiation, progression and metastasis. Genetically engineered mouse models (GEMMs) for SCLC and other high-grade lung neuroendocrine (NE) carcinomas are crucial for translational research. He presented five GEMM models representative for the entire spectrum of human high-grade NE carcinomas and also useful for the study of multistage pathogenesis and the metastatic properties of these tumours [12]. He particularly highlighted a role of cellular heterogeneity and paracrine cross-talk between different tumour cell types (NE and non-NE cells) in acquiring metastatic potential.

## Poster sessions

The conference hosted two independent poster sessions including 63 posters in total. Posters covered different aspects associated with personalised medicine, including analysis of inter- and intratumoural heterogeneity at different molecular levels in multiple tumour types, differential response to treatment and resistance. 31 participants received a travel grant of 1000E each to assist with the cost of the conference. Anne Harttrampf from the Necker Enfants Malades and Saint Anne Hospital in Paris (France) and Stefania Libreros from the Florida Atlantic University (USA) won the 2 poster prizes worth 100 € each.

## Satellite Symposia

The conference accommodated two satellite symposia presented by NanoString and Affymetrix. Both companies presented their novel technologies in personalised medicine. NanoString nCounter technology promises a high multiplex amplification-free system for detection of hundreds of targets simultaneously via digital detection. Affymetrix highlighted their new OncoScan FFPE Assay for the high resolution whole-genome copy number analysis of as little as 80ng DNA. Both technologies can be applied to challenging samples (FFPE).

## Conclusions

Precision Medicine is a novel approach for disease diagnosis and treatment that takes into account individual differences between patients. Significant improvements were made to implement targeted protocols in several cancers, however it is far from being a general clinical practice. Before the precision medicine protocols become standard we need to better comprehend the molecular drivers leading to a very strong inter- and intratumoural heterogeneity and emergence of resistance. In that respect, integrative analysis of a high-throughput techniques combined with use of patient-derived xenografts will be of a great value in the future. In addition improved understanding of tumour microenvironment and interactions with immune system will be crucial for a success of encouraging novel cancer immunotherapies and combinatory treatments. The diagnosis for precision medicine protocols needs to be straightforward and cost-effective, therefore emergence of tailored molecular tests, including analysis of circulating free DNA or circulating tumour cells appear as promising strategies. Novel clinical trials for targeted therapeutics will be essential, nevertheless much can be already learned from the existing treatments and epidemiology.

## References

[1] Alexandrov LB (2013). Signatures of mutational processes in human cancer. Nature.

[2] Ali HR, Rueda OM, Chin SF, Curtis C (2014). Genome-driven integrated classification of breast cancer validated in over 7,500 samples. Genome Biol.

[3] Amatu A (2013). Promoter CpG island hypermethylation of the DNA repair enzyme MGMT predicts clinical response to dacarbazine in a phase II study for metastatic colorectal cancer. Clin Cancer Res.

[4] Carmona FJ (2014). A comprehensive DNA methylation profile of epithelial-to-mesenchymal transition. Cancer Res.

[5] Chen ZX (2014). RNA helicase A is a downstream mediator of KIF1Bbeta tumor-suppressor function in neuroblastoma. Cancer Discov.

[6] Curtis C (2012). The genomic and transcriptomic architecture of 2,000 breast tumours reveals novel subgroups. Nature.

[7] Davies H (2002). Mutations of the BRAF gene in human cancer. Nature.

[8] Dawson SJ, Rueda OM, Aparicio S, Caldas C (2013). A new genome-driven integrated classification of breast cancer and its implications. EMBO J.

[9] Eirew P (2015). Dynamics of genomic clones in breast cancer patient xenografts at single-cell resolution. Nature.

[10] Gajjar A, Pfister SM, Taylor MD, Gilbertson RJ (2014). Molecular insights into pediatric brain tumors have the potential to transform therapy. Clin Cancer Res.

[11] Garnett MJ, Mc Dermott U (2014). The evolving role of cancer cell line-based screens to define the impact of cancer genomes on drug response. Curr Opin Genet Dev.

[12] Gazdar AF (2015). The comparative pathology of genetically engineered mouse models for neuroendocrine carcinomas of the lung. J Thorac Oncol.

[13] Girotti MR (2015). Paradox-breaking RAF inhibitors that also target SRC are effective in drug-resistant BRAF mutant melanoma. Cancer cell.

[14] Girotti MR (2013). Inhibiting EGF receptor or SRC family kinase signaling overcomes BRAF inhibitor resistance in melanoma. Cancer Discov.

[15] Girotti MR, Saturno G, Lorigan P, Marais R (2014). No longer an untreatable disease: how targeted and immunotherapies have changed the management of melanoma patients. Mol Oncol.

[16] Golebiewska A, Bougnaud S, Stieber D (2013). Side population in human glioblastoma is non-tumorigenic and characterizes brain endothelial cells. Brain.

[17] Golebiewska A, Brons NH, Bjerkvig R, Niclou SP (2011). Critical appraisal of the side population assay in stem cell and cancer stem cell research. Stem Cell.

[18] Heidorn SJ, Milagre C, Whittaker S (2010). Kinase-dead BRAF and oncogenic RAS cooperate to drive tumor progression through CRAF. Cell.

[19] Hodgkinson CL (2014). Tumorigenicity and genetic profiling of circulating tumor cells in small-cell lung cancer. Nat Med.

[20] Hovestadt V (2014). Decoding the regulatory landscape of medulloblastoma using DNA methylation sequencing. Nature.

[21] Khanna A, Pimanda JE, Westermarck J (2013). Cancerous inhibitor of protein phosphatase 2A, an emerging human oncoprotein and a potential cancer therapy target. Cancer Res.

[22] Kristensen VN, Lingjaerde OC, Russnes HG (2014). Principles and methods of integrative genomic analyses in cancer. Nat Rev Cancer.

[23] Kwong LN (2012). Oncogenic NRAS signaling differentially regulates survival and proliferation in melanoma. Nature Med.

[24] Misale S (2014a). Blockade of EGFR and MEK intercepts heterogeneous mechanisms of acquired resistance to anti-EGFR therapies in colorectal cancer. Sci Transl Med.

[25] Misale S, Di Nicolantonio F, Sartore-Bianchi A (2014b). Resistance to anti-EGFR therapy in colorectal cancer: from heterogeneity to convergent evolution. Cancer Discov.

[26] Misale S (2012). Emergence of KRAS mutations and acquired resistance to anti-EGFR therapy in colorectal cancer. Nature.

[27] Moller EK (2013). Next-generation sequencing of disseminated tumor cells. Front Oncol.

[28] Moussay E, Wang K, Cho JH (2011). MicroRNA as biomarkers and regulators in B-cell chronic lymphocytic leukemia. Proc Natl Acad Sci U S A.

[29] Noman MZ, Janji B, Kaminska B (2011). Blocking hypoxia-induced autophagy in tumors restores cytotoxic T-cell activity and promotes regression. Cancer Res.

[30] Paggetti J, Berchem G, Moussay E (2013). Stromal cell-induced miRNA alteration in chronic lymphocytic leukemia: how a minute and unavoidable cell contamination impairs miRNA profiling. Leukemia.

[31] Possik PA (2014). Parallel in vivo and in vitro melanoma RNAi dropout screens reveal synthetic lethality between hypoxia and DNA damage response inhibition. Cell Rep.

[32] Russnes HG, Lonning PE, Borresen-Dale AL, Lingjaerde OC (2014). The multitude of molecular analyses in cancer: the opening of Pandora’s box. Genome Biol.

[33] Sanchez-Laorden B (2014). BRAF inhibitors induce metastasis in RAS mutant or inhibitor-resistant melanoma cells by reactivating MEK and ERK signaling. Sci Signal.

[34] Schug ZT (2015). Acetyl-CoA synthetase 2 promotes acetate utilization and maintains cancer cell growth under metabolic stress. Cancer cell.

[35] Simpson TR (2013). Fc-dependent depletion of tumor-infiltrating regulatory T cells co-defines the efficacy of anti-CTLA-4 therapy against melanoma. J Exp Med.

[36] Stefansson OA, Villanueva A, Vidal A, Marti L, Esteller M (2012). BRCA1 epigenetic inactivation predicts sensitivity to platinum-based chemotherapy in breast and ovarian cancer. Epigenetics.

[37] Stieber D (2014). Glioblastomas are composed of genetically divergent clones with distinct tumourigenic potential and variable stem cell-associated phenotypes. Acta Neuropathol.

[38] Su F (2012). RAS mutations in cutaneous squamous-cell carcinomas in patients treated with BRAF inhibitors. N Engl J Med.

[39] Ventela S, Sittig E, Mannermaa L (2015). CIP2A is an Oct4 target gene involved in head and neck squamous cell cancer oncogenicity and radioresistance. Oncotarget.

[40] Viry E, Baginska J, Berchem G (2014). Autophagic degradation of GZMB/granzyme B: a new mechanism of hypoxic tumor cell escape from natural killer cell-mediated lysis. Autophagy.

